# Recent Advances in Imaging Sensors and Applications

**DOI:** 10.3390/s21123970

**Published:** 2021-06-09

**Authors:** Changhan Yoon, Changho Lee

**Affiliations:** 1 Department of Biomedical Engineering, Inje University, Gimhae 50834, Korea; cyoon@inje.ac.kr; 2 Department of Nanoscience and Engineering, Inje University, Gimhae 50834, Korea; 3 Department of Artificial Intelligence Convergence, Chonnam National University, 77 Yongbong-ro, Buk-gu, Gwangju 61186, Korea; 4 Department of Nuclear Medicine, Chonnam National University Medical School & Hwasun Hospital, Hwasun 58128, Korea

Recent advances in sensor technology have allowed us to develop many interesting applications and enhance the quality of human life. In particular, imaging sensors have been regarded as critical elements in achieving high-level imaging methods such as laser-based imaging, ultrasound imaging, and X-ray imaging, as well as non-destructive inspection imaging, contributing to high sensitivity, real-time display, and compact implementation. These cutting-edge sensing technologies are a crucial player in the biomedical and industrial fields.

The purpose of this Special Issue is to cover some of the recent developments in imaging sensors and their applications. The Special Issue has been co-organized by Prof. Changho Lee, Department of Nuclear Medicine, Chonnam National University Medical School, Korea, and Prof. Changhan Yoon, Biomedical Engineering, Inje University, Korea. In this Special Issue, 17 original papers and two review papers have been published [1,2,3,4,5,6,7,8,9,10,11,12,13,14,15,16,17,18,19]. Most of the papers (15 papers) are in the field of biomedical engineering and four papers are related to the field of industrial applications.

Photoacoustic imaging is an emerging technology that combines optical contrast and ultrasonic resolution. This technology allows us to visualize functional information deep inside the body with high spatial resolution, which was not possible with a pure optical imaging modality. To further increase the depth-of-field, T. P Nguyen et al. proposed a multifocal point transducer for photoacoustic microscopy [1]. This work fabricated the multifocal point transducer with seven focal points by separated spherically focused surfaces. J. Jang et al. presented a transrectal ultrasound and photoacoustic probe for prostate cancer detection [2]. The goal of this work was to develop a transrectal hybrid probe, of which the size is similar to that of the currently used transrectal ultrasound transducer. T. T. Mai et al. performed a pilot study to monitor peripheral vascular dynamic to investigate the side effects of carfilzomib using quantitative photoacoustic imaging [3]. Additionally, new tracking and visualization using fast photoacoustic microscopy have been proposed to perform the safe and accurate navigation of balloon catheters for arterial stenosis dilatation, coronary artery disease, and gastrointestinal tracking applications [4]. R. Manwar et al. proposed the photoacoustic imaging approach to estimate the maximum thickness of the skull [5].

Many research papers have been published in the field of conventional medical imaging. High-resolution imaging techniques based on synthetic aperture and plane wave have been proposed for ophthalmic and abdominal applications and their performances were evaluated through ex vivo and in vivo studies [6,7]. C. Z.-H. Ma et al. proposed a new protocol of measuring bilateral back muscle stiffness along the thoracic and lumbar spine with ultrasound imaging [8]. In this work, they ascertained that ultrasound shear-wave elastography and a tissue ultrasound palpation system produced reliable results for measuring back muscle stiffness. K. Kim et al. introduced an advanced bandwidth expander circuit composed of unique switching designs to support a wide range of the transducer with a single ultrasound imaging system [9]. In addition, two comprehensive review papers have been published in this Special Issue. One is about the recent development of super-resolution ultrasound imaging and another is about the limitation of clinical elastography diagnosis [10,11].

J. Kang et al. proposed a brain tumor classification method based on a combination of deep features and machine learning classifiers [12]. In this work, they tried to adopt the theory of transfer learning and utilized various pre-trained deep learning algorithms to acquire crucial deep features of brain magnetic resonance imaging data. For the remote sharing economy, two-photon laser scanning microscopy based on the internet of things was proposed as a remote research equipment sharing system [13]. Using the internet of things modules, they developed a web service system where data are transmitted to and received from remote users and installed in the two-photon laser scanning microscopy. S. A. Saleah et al. presented a new quad-scanner-based optical coherence tomography for visualizing the full-directional volumetric structure [14]. H. Wu et al. proposed a new approach for measuring the adjustable volumetric frequency and phase information of the human chest and abdomen surface regardless of motion artifacts [15].

For industrial applications, J. Lee et al. proposed a novel around view monitoring calibration method to avoid conventional exhaustive procedures which includes accurate positioning and estimating the calibration boards surrounding the vehicle [16]. This method only requires four pieces of random calibration information based on the correct position of individual calibrating boards. G. Lefever et al. investigated the effectiveness of elastic waves for a non-destructive testing method of cementitious samples and revealed their composites of the inner structure at the microscale [17]. K. A. Tiwari et al. presented a new analysis method of wave patterns from the macro-fiber composite transducer to overcome the limitation of accuracy issue of the previous analytical model [18]. They confirmed that the proposed model enhanced the analytical modeling for directivity pattern estimation. A multi-wavelength fluorescence LiDAR system was proposed for vegetation monitoring in forestry and agricultural applications [19]. The authors extended the system to the multi-channel fluorescence detection of laser-induced fluorescence based on the LiDAR scanning and ranging mechanism.
